# Assessment of the Analytical Performance of Three Near-Infrared Spectroscopy Instruments (Benchtop, Handheld and Portable) through the Investigation of Coriander Seed Authenticity

**DOI:** 10.3390/foods10050956

**Published:** 2021-04-27

**Authors:** Claire McVey, Una Gordon, Simon A. Haughey, Christopher T. Elliott

**Affiliations:** ASSET Technology Centre, Institute for Global Food Security, School of Biological Sciences, Queen’s University Belfast, 19 Chlorine Gardens, Belfast BT9 5DL, Northern Ireland, UK; c.mcvey@qub.ac.uk (C.M.); ugordon02@qub.ac.uk (U.G.); chris.elliott@qub.ac.uk (C.T.E.)

**Keywords:** food authenticity, near-infrared spectroscopy, chemometrics, portable, handheld

## Abstract

The performance of three near-infrared spectroscopy (NIRS) instruments was compared through the investigation of coriander seed authenticity. The Thermo Fisher iS50 NIRS benchtop instrument, the portable Ocean Insights Flame-NIR and the Consumer Physics handheld SCiO device were assessed in conjunction with chemometric modelling in order to determine their predictive capabilities and use as quantitative tools through regression analysis. Two hundred authentic coriander seed samples and ninety adulterated samples were analysed on each device. Prediction models were developed and validated using SIMCA 15 chemometric software. All instruments correctly predicted 100% of the adulterated samples. The best models resulted in correct predictions of 100%, 98.5% and 95.6% for authentic coriander samples using spectra from the iS50, Flame-NIR and SCiO, respectively. The development of regression models highlighted the limitations of the Flame-NIR and SCiO for quantitative analysis, compared to the iS50. However, the results indicate their use as screening tools for on-site analysis of food, at various stages of the food supply chain.

## 1. Introduction

The quality, safety and authenticity of food is under increasing threat due to pressures arising from the current coronavirus pandemic (COVID-19) and ongoing climate change issues. The impact of COVID-19 has led to the disruption of food supply chains, increased demand for specific food commodities, and resulted in significant wastage of other food products. The price of food remained relatively stable in the first few months of the pandemic. However, the potential for increased food prices in the future is expected [1]. This instability within the food supply system, merged with a reduced level of surveillance, has the potential to allow low-quality, unsafe and fraudulently produced commodities to enter the food chains. The recent seizure of two shipments of horsemeat destined for the EU markets is a reminder of the vulnerability within food chains [2]. It is evident that there is a need for analytical testing approaches that can be implemented at various stages of the food supply chain in order to mitigate food quality, safety and authenticity risks.

Traditional methods employed for food safety, quality and authenticity analysis, such as mass spectrometry [3], DNA analysis [4] and culturing techniques [5], are generally expensive, time consuming, non-conducive to on-site analysis and require highly trained personnel. To overcome these issues, vibrational spectroscopy (near infrared, mid-infrared and Raman) based approaches have been used due to the fast, non-invasive and simultaneous analysis of different chemical and physical parameters [6]. Due to advances in micro-technologies, such as Micro-Electro-Mechanical Systems (MEMS), Micro-Opto-Electro-Mechanical Systems (MOEMS), e.g., digital mirror device (DMD™) and Linear Variable Filters (LVFs), near-infrared spectroscopy (NIRS) has transitioned from benchtop/laboratory equipment to a highly portable, low-weight and rugged device [7,8,9]. The incorporation of these technological advances makes NIRS handheld or portable devices more cost effective over their mid-infrared (MIR) and Raman counterparts. These features make them applicable to widespread use, particularly on-site or on-line analysis. This has been demonstrated through the incorporation of NIRS handheld devices into process analytical technology (PAT), due to the potential for real-time monitoring and modelling of food constituents during the manufacturing process [10].

The advantages and wide applicability of NIRS have accelerated the commercial development of handheld devices. A comparison of commercially available handheld NIR spectrometers is shown in Table 1. One of the most widely used and commercially available, handheld NIR spectrometers is the Viavi MicroNIR 1700ES (formerly JDSU, Santa Rosa, CA, USA). It incorporates LVF as the monochromator and has an array detector that covers the wavelength range from 950 to 1650 nm. It is the incorporation of the LVF that affords the MicroNIR 1700ES its low cost, good spectral resolution, compact design and low power consumption. New system developments have focused on the incorporation of single-point detectors rather than array detectors, in a bid to further reduce the cost of the device. Texas Instruments have developed DLP NIRscan Nano EVM (Dallas, TX, USA), which replaces the traditional linear array with digital light processing (DLP) digital micromirror device (DMD) in conjunction with a single-element detector. The technology affords low cost, compact size, higher performance (uses a larger single-point 1 mm detector in comparison to linear array) and improved functionality (e.g., programmable spectral filters and sampling techniques) over traditional architectures. The SCiO (Consumer Physics, Israel) uses a silicon photodiode array (PDA) detector that is sensitive in the range of 740–1070 nm. The wavelength range is limited in comparison to other devices that require indium gallium arsenide (InGaAs) detectors, but the PDA detector is less expensive. Recently, Si-Ware Systems (Cairo, Egypt) developed a MEMS-based FT-NIRS device that contains a single-chip Michelson interferometer with a monolithic optoelectro-mechanical structure. The incorporation of MEMS allows for the simultaneous measurement of various materials over a wider range, and at the higher end of the NIR spectrum (1150 to 2500 nm), and for the adjustable resolution of the spectrometer. Spectral Engines (Helsinki, Finland) also incorporate MEMS technology into their miniaturised NIRONE spectrometers, alongside a Fabry-Perot Interferometer, which is a fully programmable, optical filter. A drawback is the requirement for five spectrometers to cover the NIR wavelength region 1350 to 2450 nm (S1.4-EVK; 1100–1350 nm, S1.7-EVK; 1350–1650 nm, S2.0-EVK; 1550–1950, S2.2-EVK; 1750–2150 and S2.5-EVK; 2000–2450 nm). For a more in-depth overview of miniaturised NIR technology, the reader is prompted to the following review [11].

Due to these advances and advantages of portable or handheld NIRS devices, research has been carried out to determine the applicability of commercial devices in the area of food safety, quality and authenticity analysis. Applications include (i) authenticity testing [12,13,14,15,16,17,18], (ii) post-harvest quality of fruit [19], (iii) pork meat composition (fat, moisture and protein) [20], (iv) determination of sucrose levels in infant cereals [21], (v) acrylamide content in potato chips [22], and (vi) nutritive composition of compound feedstuffs [23]. The low cost, non-invasive measurement, compact size and ease of use of NIRS handheld and portable devices meet the requirements for on-site or on-line analysis. Thus, further investigations are warranted into the analytical performance of such devices for food quality, safety and authenticity screening. Current research has compared the performance of a variety of commercially available portable and handheld devices against one another [22,23,24] and against benchtop instrumentation [10,17,18,25,26,27,28] for food analysis. To the best of the author’s knowledge, only one publication has evaluated the analytical performances of a NIRS benchtop instrument, handheld and portable device [16]. This research focused on the authenticity of honey origin using three NIRS instruments, which are different to those used in the current study. The research focused on a small sample size and determined predictive capabilities only. To the best of the author’s knowledge, no research has been conducted in the area of coriander authenticity via handheld or portable NIRS. It is evident that more research is required to assess the performance of handheld and portable NIRS instruments in comparison to their benchtop counterparts as a means to improve confidence in the application of these types of instrumentation in field-based applications.

Therefore, the aim of this work was to assess the analytical performance of three commercially available NIRS instruments, differing in wavelength range, resolution and portability. A benchtop NIRS (iS50, Thermofisher Scientific, Dublin, Ireland), a portable NIRS (Flame-NIR, Ocean Insight, USA) and a handheld NIRS (SCiO, Consumer Physics, Israel) were compared to assess their analytical performance quantitatively and qualitatively. These devices were chosen based on their differing specifications, but also their individual utilisation in the area of food authenticity analysis [29,30,31,32,33,34]. To the best of the author’s knowledge, there is no reported authenticity methods developed using the Flame-NIR. In contrast, methods using the SCiO device have increased over the last number of years [32,33,34]. However, no methods have been developed for herb and spice authenticity analysis. With the increasing need for rapid, on-site analysis of food, it is anticipated that these findings will provide further knowledge of the accuracy and precision of commercially available portable or handheld NIR spectrometers in comparison to their benchtop counterparts. We assess and compare the actual performances of each of the devices for the determination of coriander seed authenticity, which can differ from theoretical indications, due to the spectral variation of food samples. Furthermore, the assessment of the SCiO cloud-based software, The Lab, was conducted to determine the analytical processing power in combination with the SCiO handheld device. Due to the ubiquitous use in Indian, Asian, Middle Eastern and Mediterranean cuisines and potentional vulnerability to fraud [35], coriander seed was chosen for the method development.

## 2. Materials and Methods

### 2.1. Sample Collection and Preparation

Two hundred, whole coriander seed samples with highest-grade quality (AAA rated) provenance and authenticity, of Indian origin, were obtained from a reputable supplier. Potential adulterants were identified; salt and sawdust were purchased at a local retailer (Asda) and starch was obtained from Sigma Aldrich, Pool, UK (CAS number: 9005-25-8). All samples were stored in sealed containers, at ambient temperature and away from direct light. Prior to spectroscopic analysis, all samples were ground to a homogenous powder using a ball mill (Planetary Ball Mill: PM-100 Retsch, Hann, Germany). Approximately 5.0 g of coriander seed was added to the grinding jars and milled at 500 rpm for five minutes. To prepare the adulterated samples, 10 authentic coriander samples were chosen (Excel random generator) and spiked at levels of 10, 20 and 30 g/100 g with salt, sawdust or starch. The concentrations used in this study were chosen based on economically relevant levels for adulteration. In total, 90 spiked samples were prepared.

### 2.2. Near-Infrared Spectroscopy

Three NIRS instruments were chosen for the current study. These included benchtop equipment, iS50 (Thermo Fischer Scientific, Dublin, Ireland), Flame-NIR (Ocean Insight, USA), a portable NIRS connected to a laptop, and a handheld SCiO device (Consumer Physics, Tel Aviv, Israel) with Bluetooth connectivity to a smartphone device. All samples were analysed in the milled form at ambient temperature, three replicates of each sample were measured and the average was calculated prior to data processing.

The Thermo iS50 NIRS benchtop instrument was operated in the spectral range of 833–2500 nm (12,000–4000 cm^−1^) with 32 scans and a resolution of 8 cm^−1^. The samples were poured into the sample cup (diameter: 3.2 cm, height: 1.5 cm) spinner on the integrating sphere module of the instrument. The resulting spectral data was analysed using SIMCA 15 chemometric Software (Sartorius, Sweden) and TQ Analyst 8 (Thermo Fisher Scientific, Dublin, Ireland).

The portable Flame-NIR functions in the wavelength range of 934–1660 nm (10,707–6250 cm^−1^). Prior to sample analysis, the device was turned on for thirty minutes to allow the light source to warm up. Prior to the first measurement, device calibration was carried out using two Spectralons (99% and 0% reflectance) and this was subsequently updated every 10 min thereafter. The samples were poured into a Petri dish and placed over the scan window. The Petri dish has no effect on the spectra. Three measurements were collected in the reflectance mode with manual rotation in between replicates. The spectra were downloaded from the Ocean View user interface and analysed using SIMCA 15 software and TQ analyst.

The handheld SCiO operates in the spectral range of 740–1070 nm (13,514–9346 cm^−1^). The device was calibrated prior to analysis via insertion of the source and detector downwards into the outer protective case where the calibration module is embedded. The calibration process is initiated through the application and this was repeated every ten minutes during the analysis. In order to warm up the light source, three scans were initiated. For sample analysis, a Petri dish was used to hold the sample and this was placed on top of the SCiO device. The Petri dish has no effect on the spectra. The samples were measured three times in diffuse reflectance mode, with manual rotation of the Petri dish in between scans. The spectra were analysed with the cloud-based software, The Lab (SCiO), as well as with SIMCA 15 chemometric software.

### 2.3. Multivariate Data Analysis

#### 2.3.1. Spectral Pre-Treatment

Spectra obtained on the Thermo iS50 NIRS, Flame-NIR and SCiO were exported and evaluated using SIMCA 15 chemometric software. Prior to spectral processing, the data obtained from each instrument was averaged. Pareto scaling was used throughout, allowing for a reduction in the relative significance of large values, whilst at the same time maintaining the structure of the data, by using the square root of the standard deviation as the scaling factor [36]. Different pre-processing strategies were assessed; data was pre-treated with standard normal variate (SNV), first-order derivatives (1DER), Savitzky–Golay (SG) smoothing or a mixture thereof in order to remove irrelevant light scattering from the spectra and to separate overlapping peaks.

Spectra recorded using the SCiO device were also evaluated using the online, cloud-based, The Lab software (Consumer Physics). This software works specifically with the SCiO device and aims to integrate spectral collection and chemometric analysis to provide the user with a rapid result using publically available databases or privately created ones. Various pre-processing methods were assessed using The Lab software in custom mode, which allows for the selection of specific algorithms including SNV and first and second-order polynomial smoothing with a window of 35 (1DER, 2DER). The Lab software also features log transformation of the spectra and subtraction of the average or minimum spectra.

For regression analysis using TQ Analyst 8 (Thermo Fisher Scientific), various spectral pre-processing techniques were assessed including log transformations and first and second-order derivatives (1DER and 2DER). Savitzky–Golay (SG) smoothing was also assessed in conjunction with the other pre-processing techniques. Due to compatibility issues, data obtained on the SCiO device was unable to be uploaded to TQ Analyst for regression analysis.

#### 2.3.2. Classification Models

For chemometric model development and validation, the samples were split into a reference set which consisted of 192 samples and a validation set, which consisted of 98 samples which had not been used in the construction of the model (using random number generator on Excel). The reference and validation sets represented authentic coriander seeds (67%), starch adulterated (11%), salt adulterated (11%) and sawdust adulterated (11%).

Chemometric model development was carried out using SIMCA 15 software (SIMCA, Sartorius, Sweden), with classification models built using Partial Least Squares Discriminant Analysis (PLS-DA) or orthogonal PLS-DA (OPLS-DA). Multiclass and two-class models were created using the spectral data, focusing on either authentic and starch, salt, sawdust adulterated or authentic and all adulterated samples. The models were validated internally via cross-validation, with 1/7th removed and used as the validation set, and remaining 6/7th used to build the model. The prediction error was calculated for each validation set and this procedure was repeated for all subsets. The predictability of the models was also validated using the external validation set (data not used in model development). A ‘cut-off’ value was determined using the predicted scores obtained from SIMCA 15 software during the classification. The highest prediction score, which encompassed the maximum correct predictions (authentic coriander and adulterant samples), was calculated. This allowed for the comparison of sensitivity and specificity of each model.

For data analysis using The Lab software, Random Forest (RF) algorithm was used to develop classification models to predict the authenticity of the coriander samples. The models were tested using the external validation set and the predictability of the models were assessed. The Lab software does not offer any information about the internal validation of their models.

#### 2.3.3. Regression Models

The same reference and validation sets that were used in the development of classification models were used in the construction of regression models. Each potential adulterant was assessed separately alongside the authentic coriander seed samples. The pre-processed data was uploaded onto TQ Analyst 8 software (iS50 NIRS and Flame-NIR data) or The Lab (SCiO data only) software. Using the spectral data obtained from TQ Analyst, regression models were created using Principal Component Regression (PCR) and Partial Least Squares Regression (PLS-R) algorithm. The validation samples were used to test the models. For the analysis using The Lab (SCiO data only) cloud-based software, Partial Least Squared Regression (PLS-R) algorithm was used to create the models. To evaluate the analytical performance of the regression models, several quality parameters were determined. The coefficient of determination (R^2^), a measure of the linearity; the root mean square error of cross-validation (RMSECV) and root mean square error of prediction (RMSEP) were calculated to determine the accuracy of the established models. Furthermore, regression models developed using TQ Analyst were evaluated in terms of their limit of detection (LOD) and limit of quantification (LOQ). The LOD is the lowest analyte concentration which can be reliably detected for a given analytical procedure (LOD = 3.29σ of the authentic samples) and the LOQ is the lowest concentration which can be detected (LOQ = 10σ of the authentic samples) [37].

## 3. Results and Discussion

### 3.1. Near-Infrared Spectroscopy

**Figure 1:**
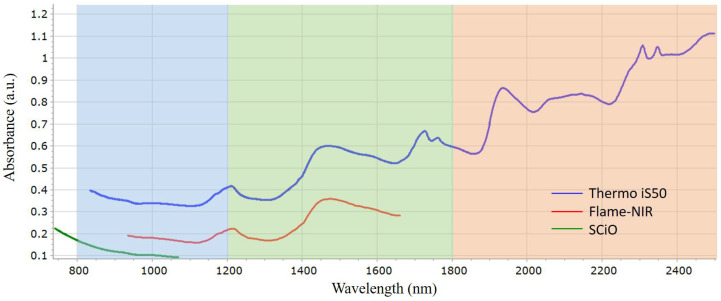
Average spectrum of authentic coriander seed samples assessed using the Thermo iS50 (blue), Flame-NIR (red) and SCiO (green). The background colors represent the three NIR regions, blue (region 1), green (region 2) and orange (region 3).

NIR spectra consists of overtones and combination bands that are usually overlapping and therefore complex. Figure 1 demonstrates the average spectrum for authentic coriander seed samples obtained on the three NIRS instruments, with the different NIR regions highlighted. Region 1/“Herschel” region ranges from 800 to 1200 nm (blue), region 2 comprises the range between 1200 and 1800 nm (green) and region 3 encompasses the region of 1800–2500 nm (orange). Each device is operational over varying regions and this potentially influences the information that can be determined. Electronic transitions, overtones and combination bands are observed in region 1, first overtones and combination bands are demonstrated in region 2 and region 3 mostly displays combination modes [38].

The iS50 benchtop NIR encompasses the spectral range from 833 to 2500 nm (12,000–4000 cm^−1^). Thus, the iS50 utilises all three NIR regions and, in theory should provide the most accurate information in comparison to the handheld and portable devices. However, due to the number of combination bands over the three regions, data interpretation can be complex. In contrast, the handheld and portable devices operate over a shorter range. Spectra obtained from the Flame-NIR utilises the spectral range between 934 and 1660 nm (10,707–6250 cm^−1^), which corresponds to region 1 and 2 of the NIR spectrum. The SCiO operates in the spectral range of 740–1070 nm (13,514–9346 cm^−1^) which encompasses region 1 and, interestingly, reaches into the visible part of the electromagnetic spectrum. This could be of benefit if the adulterant influences the sample color (i.e., lightens or darkens), which is the case in terms of the current study. The low wavelength range of region 1 is also beneficial in terms of sample penetration, which is important in non-homogenous samples. However, lower wavelengths demonstrate low band intensities due to their association with second and third overtones [24]. These differences in spectral range ultimately influence the predictability and robustness of the developed chemometric models.

### 3.2. Model Development and Validation

#### 3.2.1. Model Development Using SIMCA 15 Software

PLS-DA and OPLS-DA supervised models were developed using SIMCA 15 software. Various pre-processing techniques were applied to the spectral data to assess the predictability of the models (Table 2). Both the iS50 and Flame-NIR correctly predicted 100% of the adulterated samples using all of the models (M1–16; Table 2). The best predictions were determined using the benchtop instrument, the iS50, in which correct predictions of 100% were obtained for both authentic coriander and the adulterants (M3–7, M9–11, M13 and M16). This was expected due to the wavelength coverage of the instrument, which encompasses the three NIR regions. M11, a PLS-DA multi-class model with data transformed with SNV, was deemed to be the best model overall based a high cut-off value and R^2^ of 0.99 and Q^2^ of 0.70. The high R^2^ and Q^2^ values indicate that the model can explain most of the spectral variation and that it has good predictability. M11 consisted of 3 factors that explained 99.7% of the variation (Appendix A).

The best model obtained for the portable Flame-NIR instrument was a two-class, PLS-DA model with no spectral pre-processing (M1). This model resulted in correct predictions of 98.5% and 100% for authentic coriander and adulterant samples, respectively. In this case, a R^2^ of 1.0 and a Q^2^ of 0.78 was determined. Using M1, 99.9% of the variation was explained using 5 factors (Appendix A).These results are comparable to the benchtop iS50 instrument and demonstrate the applicability of the NIR region 2 to the current investigation. Thus, the Flame-NIR demonstrated very good potential as a portable device for authenticity analysis.

**Figure 2:**
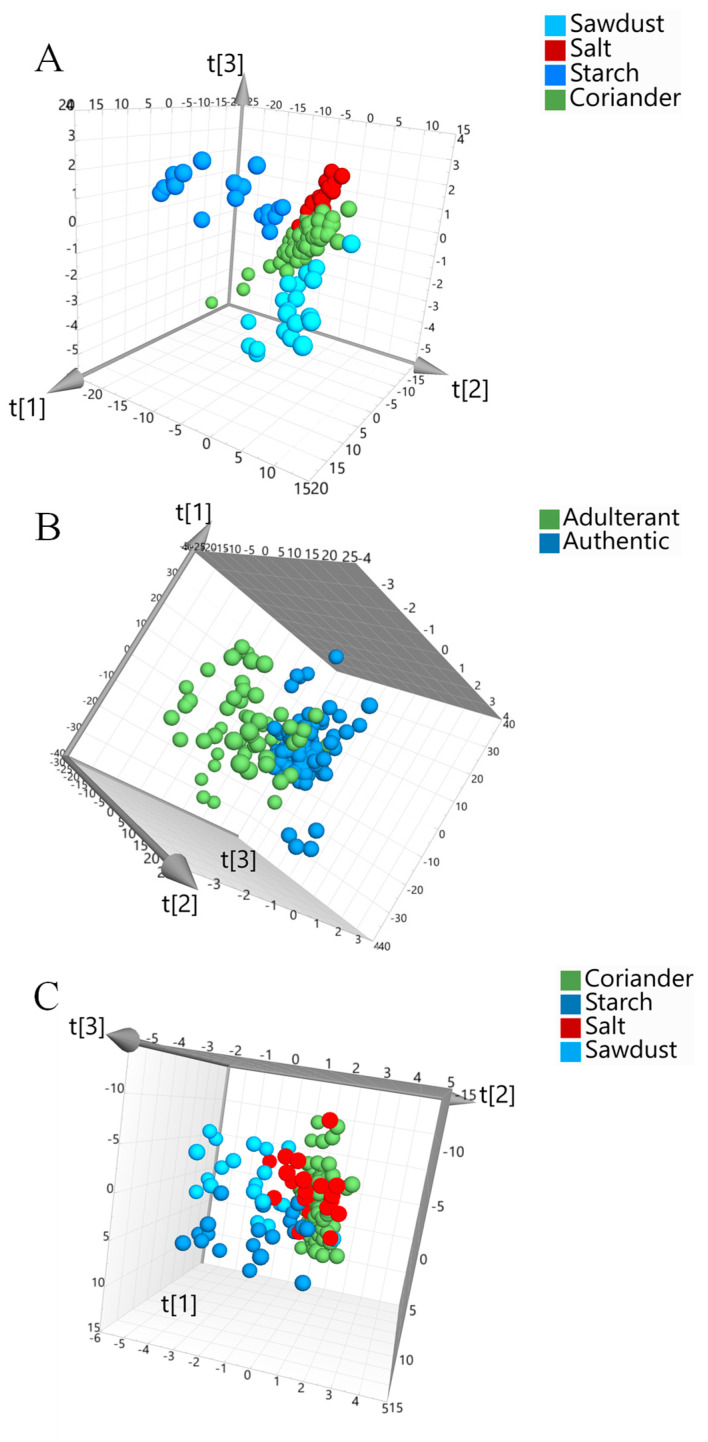
The best classification models obtained for each NIRS instrument developed using SIMCA 15 software; (**A**) Spectra obtained from the Thermo iS50 benchtop instrument; a multiclass, PLS-DA model with SNV pre-processing applied (R^2^ = 0.99, Q^2^ = 0.70). (**B**) The portable Flame-NIR obtained data; a PLS-DA, two-class model with no spectral pre-processing applied (R^2^ = 1.0, Q^2^ = 0.78). (**C**) Data obtained from the handheld SCiO device, pre-processed with SNV and 1DER; a PLS-DA, multiclass model (R^2^ = 0.99. Q^2^ = 0.52).

The majority of the models were able to predict 100% of the adulterant samples using data obtained on the handheld SCiO. The correct predictions for the authentic coriander samples were generally lower in comparison to the results obtained for both the benchtop iS50 and the portable Flame-NIR. However, correct predictions of over 90% were achieved. This was expected due to the limited wavelength range of the SCiO device. The model that resulted in the greatest correct predictions was a PLS-DA multiclass model that used SNV and 1DER transformed data (M13). The model achieved correct predictions of 95.6% for authentic coriander samples and 100% for the adulterant samples, with a R^2^ and Q^2^ value of 0.99 and 0.52. A lower Q^2^ value was achieved in comparison to that obtained for the iS50 and Flame-NIR best models, this corresponds with the lower predictability found during external validation for authentic samples. M13 used four factors capable of explaining 99.6% of the variation (Appendix A). Although the SCiO demonstrated lower predictability of the authentic coriander samples, the results are adequate for use as a screening approach, where samples determined to be ‘adulterated’ will undergo confirmatory analysis. The best models for each instrument are shown in Figure 2. Clear separation between the authentic coriander and adulterant samples is observed.

#### 3.2.2. Model Development Using the Lab Software (SCiO Only)

The Lab software was also used to develop models from the spectral data obtained using the handheld SCiO device (Table 3). Interestingly, predictions obtained using The Lab software favored the determination of authentic coriander over the adulterated samples. This differs from the prediction models developed using SIMCA 15 software, were predictability was greater for the adulterants. This is a limitation of The Lab software, as wrongly predicted ‘adulterated’ samples will be classified as ‘authentic’, and subsequently allowed to enter the food chain. Nonetheless, the best predictions were obtained for M1 (Table 3), a two-class model built with spectral data which had not undergone any pre-processing. Correct predictions of 99% and 89% were obtained for authentic coriander samples and adulterants, respectively. Compared to the best model obtained from the SIMCA 15 analysis of the SCiO spectral data (M13; 95.6% and 100% correct predictions for authentic oregano and adulterants, respectively), correct prediction of the adulterants had decreased. These variations are probably due to the differences in algorithms used in the software. The Lab software provides a performance value (F1) which indicates the accuracy of the models to detect authentic coriander or adulterated samples. For M1 the F1 value was the highest at 0.966. The Lab software has limited statistical development opportunities as it is consumer-orientated. This has both positive and negative implications, as it is easily accessible to many users but does not allow a more in-depth development of statistical parameters. Based on this finding, SIMCA 15 software is preferable for the development of prediction models using spectral data from the SCiO device. However, due to restrictions on the software, the assessment of spectral data from other instruments is prohibited. Thus, it not known whether this finding would be consistent across other instruments.

#### 3.2.3. Regression Models Developed Using TQ Analyst Software (iS50 and Flame-NIR)

Regression (PCR) models were established and externally validated for each of the three potential adulterants using TQ Analyst 8 (Thermo Fisher Scientific, Waltham, MA, USA). Spectral data obtained from the benchtop iS50 and portable Flame-NIR was uploaded onto the software, and various pre-processing parameters were assessed. The iS50 instrument outperformed the Flame-NIR device in terms of linearity, accuracy, limits of detection and quantification and recovery (Table 4). The coefficient of determination (R^2^) was above 0.9 for all models developed using the iS50-derived data, over all of the adulterants tested (salt, starch and sawdust). The regression models developed for starch demonstrated particularly good linearity (between 0.99 and 1.0). These results indicate good correlation between the spectral and reference data. A low root mean square error of cross-validation (RMSECV) (0.33–4.33) and root mean square error of prediction (RMSEP) (0.31–4.59) was determined for the regression models developed using the iS50 derived spectral data, indicating good accuracy across all adulterants tested. The regression models for starch in particular demonstrated a low RMSECV and RMSEP, with the lowest values of 0.33 and 0.31 obtained using M6. This highlights the potential of the models for the quantification of starch. The LOD and LOQ coincided with these findings. The best starch regression model had the lowest LOD and LOQ of 0.6% and 1.9% (M6), respectively. The LOD and LOQ for salt and sawdust were slightly higher than that found for starch. The best regression model demonstrated a LOD and LOQ for salt of 3.5% and 10.6% (M2), respectively and 2.8% and 8.4% for sawdust (M7). The recoveries were generally between 90% and 100% for all of the models with regards all adulterants, with a few outliers. These findings demonstrate good accuracy and robustness of the models using spectral data from the benchtop iS50.

The regression models developed using spectral data from the portable Flame-NIR device demonstrates a lower coefficient of determination (R^2^) for all the adulterants in comparison to the regression models developed using spectral data from the iS50. This finding suggests that these models are less robust and may require more samples to improve the accuracy. Furthermore, the RMSECV (1.92–5.39) and RMSEP (1.65–5.39) were greater in comparison to the regression models developed using spectral data from the iS50. As the iS50 covers a larger spectral range, more information is provided between 1700 and 2500 nm, which might explain the improved accuracy of the model. Similar to the results found for the iS50 regression models, the analysis of starch adulteration using the Flame-NIR demonstrated a lower RMSECV and RMSEP of 1.92 and 1.65 (M2), respectively, in comparison to salt and sawdust. These models in particular are robust and demonstrate good accuracy based on the high fitting and prediction efficiency. The LOD and LOQ for starch using the regression models developed from Flame-NIR spectral data was 5.6% and 17.5%, respectively (M2). The lowest LOD and LOQ determined from the sawdust regression models were calculated at 7.6% and 33.1%, respectively (M7). Interestingly, the LOD and LOQ for salt was lower than that found for starch and sawdust, with a level of 4.8% and 14.6% determined (M5). This is of significance due to the inability of salt to absorb NIR light. Nonetheless, it is likely that the developed models are capable of recognising differences between the samples, not specifically the addition of salt. This correlates with the recovery values determined for salt, with over recovery noted at 10% and under recovery calculated at 20% and 30%. In general, the recoveries were poor for the Flame-NIR regression models in comparison to those obtained from the iS50 regression models. Again, the extended wavelength range on the iS50 instrument can explain this difference.

Partial Least Squares Regression (PLS-R) models were also developed using TQ Analyst and spectral data from the iS50 and Flame-NIR. The best models are shown in Table 5. The best PLS-R models developed using spectral data from the iS50 demonstrated a comparable R^2^, but higher RMSECV, RMSEP, LOD and LOQ values in comparison to the developed PCR models with regard to all adulterants (M6; starch, M2; salt, M7; sawdust, Table 4). Interestingly, all of the best PLS-R models developed using the Flame-NIR spectral data underwent no pre-processing. In the case of the processed spectral data, there was poor correlation between the spectral and reference data, possibly due to overfitting of the data. The best model developed using Flame-NIR spectral data for salt determination showed improved LOD and LOQ over the best PCR model (Table 4, M5). The best regression model for starch determination demonstrated comparable results with the best PCR model (Table 5, M2). However, sawdust quantification improved using the best PLS-R model in comparison to the best PCR model (Table 4, M5).

#### 3.2.4. Regression Models Developed Using the Lab Software (SCiO Only)

Due to the set-up of the SCiO device and the web-based, The Lab software, data could not be transferred to TQ Analyst. Thus, PLS-R models were developed on The Lab software directly using spectral data obtained from the SCiO device. Various data pre-processing transformations were applied. However, The Lab software is limited by the available statistical methods. The results are displayed in Table 6. Generally, the R^2^, RMSECV and RMSEP are consistent between the different models for each individual adulterant. Again, the regression models for starch stand out due to a higher R^2^ and lower RMSECV and RMSEP value in comparison to the other adulterants. The spectral range of the SCiO, which enters into the visible part of the spectrum, may explain this finding. The lower R^2^ value and higher RMSECV and RMSEP value for the salt regression models coincides with the previous findings in Table 4 and Table 5, and can be explained by the poor absorption of NIR light by salt. The Lab software has a set criteria to validate the developed models. This prohibited the external validation of the PLS-R models as more samples were required and limited the calculation of other parameters such as LOD, LOQ and recovery. In comparison to the best PLS-R models developed using iS50 derived data, the linearity, predictability and fit (R^2^, RMSEP and RMSECV) is poor. However, the PLS-R models developed using Flame-NIR derived data are comparable. Nonetheless, it is evident from these preliminary findings that the SCiO has the potential to be useful in starch quantification.

## 4. Conclusions

Three NIRS instruments were investigated to evaluate their performance as an analytical tool for food authenticity analysis. The three instruments, with varying specifications, namely different wavelength ranges and portability, were assessed based on classification and regression model development via chemometric software (use as a qualitative and quantitative tool). This included the development of prediction models using SIMCA 15 and The Lab software. The best predictions were determined using the benchtop iS50 derived spectral data, with 100% correct predictions for authentic coriander seeds and adulterated samples. The models developed using the portable Flame-NIR spectral data also demonstrated good predictability with 98.5% and 100% of authentic coriander and adulterant samples correctly identified. The best model developed using spectral data from the handheld SCiO correctly predicted 95.6% of the authentic coriander samples and 100% of adulterated samples. Although the predictive performance of the Flame-NIR and SCiO is lower relative to the iS50, both present adequate results for use as a screening technique. As the best models for both instruments can correctly predict 100% of the adulterated samples, any sample wrongly identified as adulterated will go through confirmatory analysis. Although SIMCA analysis of the spectral data demonstrated high predictability, analysis of The Lab software was necessary to fully assess the on-site usability of the SCiO device. Considering the attributes of each instrument, in terms of sensitivity, robustness, cost, usability and portability, both the Flame-NIR and SCiO devices demonstrate an excellent analytical tool for on-site food integrity screening.

## Figures and Tables

**Table 1:** Overview of a selection of commercially available handheld NIRS.

Spectrometer	Spectral Resolution (nm)	Measurement	Detector	Spectral Resolution	Software	Dimensions	Weight
MicroNIR 1700 ES (Viavi)	950–1650	Diffuse reflection, transmission, or transflection	128 pixel InGaAs photodiode array	12.5 nm at 1000 nm	VIAVI MicroNIR Pro software suite^a^ (data acquisition, calibration and method development, user management, and real-time prediction)	50 ×45 mm	64 g
DLP NIRscan Nano EVM (Texas Instruments)	900–1700	Diffuse reflection	1 mm single-pixel InGaAs uncooled linear array	10 nm	DLP NIRscan Nano software ^b^ (graphical user interface only)	62 × 58 × 36	54 g
SCiO (Consumer Physics)	740–1070	Diffuse reflection	Silicon photodiode array	28 nm average across its working spectral region of 740–1070 nm	TheLab ^a^ (data acquisition, calibration and method development, user management, and real-time prediction)	67.7 × 40.2 × 18.8 mm	35 g
NeoSpectra Micro (Si-Ware)	1350–2550	Diffuse reflectance	Single uncooled InGaAs photodetector	16 nm at 1500 nm	SpectroMOST software ^bc^ (graphical user interface only). Software development kit is available for development of application software	60 × 30 × 40 mm inclusive of Raspberry Pi board)	17 g
NIRONE Sensor S (Spectral Engines)	Various: 1100–1350 1350–1650 1550–1950 1750–2150 2000–2450	Diffuse reflectance	Single element InGaAs	Various depending on the spectral range: 12–28 nm	SensorControl ^b^ (graphical user interface only)	25 × 25 × 17.5 mm	15 g

^a^ Data acquisition, calibration and method development, user management, and real-time prediction. ^b^ Graphical user interface (GUI)**.** ^c^ Software development kit is available for development of application software.

**Table 2:** Predictability of the two-class and multiclass models developed and externally validated (*n* = 98) using SIMCA 15 software.

				Predictability					
				Thermo Is50		Flame-NIR		SCiO	
Type	ID	Model	Pre-Processing	Coriander	Adulterants	Coriander	Adulterants	Coriander	Adulterants
2-CLASS	M1	PLS-DA	None	97.1%	100.0%	98.5%	100.0%	92.3%	96.7%
	M2	OPLS-DA	None	97.1%	100.0%	95.6%	100.0%	92.6%	96.7%
	M3	PLS-DA	SNV	100.0%	100.0%	98.5%	100.0%	92.6%	96.7%
	M4	OPLS-DA	SNV	100.0%	100.0%	97.1%	100.0%	91.1%	100.0%
	M5	PLS-DA	SNV + 1DER	100.0%	100.0%	95.6%	100.0%	91.1%	100.0%
	M6	OPLS-DA	SNV + 1DER	100.0%	100.0%	97.1%	100.0%	91.1%	100.0%
	M7	PLS-DA	SNV + 1DER + SG	100.0%	100.0%	94.1%	100.0%	91.1%	100.0%
	M8	OPLS-DA	SNV + 1DER + SG	98.5%	100.0%	95.6%	100.0%	91.1%	100.0%
MULTICLASS	M9	PLS-DA	None	100.0%	100.0%	97.1%	100.0%	92.6%	100.0%
	M10	OPLS-DA	None	100.0%	100.0%	97.1%	100.0%	91.2%	100.0%
	M11	PLS-DA	SNV	100.0%	100.0%	95.6%	100.0%	91.2%	100.0%
	M12	OPLS-DA	SNV	97.1%	100.0%	95.6%	100.0%	91.2%	100.0%
	M13	PLS-DA	SNV + 1DER	100.0%	100.0%	94.1%	100.0%	95.6%	100.0%
	M14	OPLS-DA	SNV + 1DER	98.5%	100.0%	94.1%	100.0%	94.1%	100.0%
	M15	PLS-DA	SNV + 1DER + SG	94.1%	100.0%	94.1%	100.0%	91.2%	100.0%
	M16	OPLS-DA	SNV + 1DER + SG	100.0%	100.0%	94.1%	100.0%	91.2%	93.3%

**Table 3:** Predictability of the two-class and multiclass models developed and externally validated (*n* = 98) on the handheld SCiO device, using The Lab software.

			Predictability	
Type	ID	Pre-Processing	Coriander	Adulterants
2-CLASS	M1	None	99.0%	89.0%
	M2	Log	100.0%	88.0%
	M3	SNV	96.0%	90.0%
	M4	1DER	100.0%	86.0%
	M5	2DER	96.0%	91.0%
	M6	SNV + 1DER	98.0%	82.0%
	M7	SNV + 2DER	99.0%	82.0%
	M8	Log + SNV + 1DER	97.0%	85.0%
	M9	Log + SNV + 2DER	99.0%	84.0%
MULTICLASS	M10	None	100.0%	82.0%
	M11	Log	100.0%	85.0%
	M12	SNV	100.0%	63.0%
	M13	1DER	99.0%	87.7%
	M14	2DER	99.0%	88.7%
	M15	SNV + 1DER	100.0%	75.3%
	M16	SNV + 2DER	98.0%	80.0%
	M17	Log + SNV + 1DER	98.0%	79.3%
	M18	Log + SNV + 2DER	97.0%	74.6%

**Table 4:** Statistical parameters determined from PCR models developed using TQ Analyst software with spectral data obtained from the iS50 and Flame-NIR instruments.

		Salt								Starch								Sawdust							
							Recovery (%)								Recovery (%)								Recovery (%)		
	Model	R^2^	RMSEC	RMSEP	LOD (%)	LOQ (%)	10.00	20.00	30.00	R^2^	RMSEC	RMSEP	LOD (%)	LOQ (%)	10.00	20.00	30.00	R^2^	RMSEC	RMSEP	LOD (%)	LOQ (%)	10.00	20.00	30.00
iS50	1.00	0.94	2.56	2.07	4.80	14.70	100.05	92.89	90.99	1.00	0.46	0.47	1.10	3.30	106.97	98.28	99.67	0.96	4.33	4.59	3.50	10.50	106.59	91.89	86.19
	2.00	0.95	2.30	1.82	3.50	10.60	132.25	98.75	85.99	1.00	0.65	0.34	0.70	2.20	103.12	97.98	99.82	0.96	2.07	3.34	4.20	12.70	109.15	93.43	94.33
	3.00	0.91	3.02	2.74	6.60	20.10	110.28	88.30	86.30	0.99	0.92	1.04	2.40	7.20	89.06	91.72	99.01	0.92	2.12	3.44	5.40	16.50	106.81	84.80	76.22
	4.00	0.94	2.56	2.07	4.80	14.50	100.05	92.89	90.99	1.00	0.46	0.47	1.10	3.30	106.97	98.28	99.67	0.96	2.96	3.96	3.46	10.50	106.59	101.21	84.19
	5.00	0.94	2.56	2.07	4.80	14.50	106.97	98.28	99.67	1.00	0.46	0.47	1.10	3.30	106.97	98.28	99.67	0.96	2.07	3.34	3.50	10.50	106.58	101.21	85.33
	6.00	0.95	2.31	1.83	3.50	10.60	131.83	98.69	85.92	1.00	0.33	0.31	0.60	1.90	103.06	98.06	99.81	0.98	2.07	3.34	4.50	13.70	97.09	108.38	88.55
	7.00	0.91	2.95	2.67	6.10	18.60	94.23	85.53	89.79	1.00	0.37	0.39	0.80	2.40	97.98	96.65	100.17	0.97	1.51	2.99	2.80	8.40	101.83	102.96	91.44
	8.00	0.94	2.56	2.07	4.80	14.50	100.05	92.89	90.99	1.00	0.46	0.47	1.90	3.30	106.97	98.28	99.67	0.96	1.73	3.15	4.70	14.40	106.58	101.21	84.47
Flame NIR	1.00	0.88	3.65	3.41	5.10	15.40	137.45	77.23	76.22	0.96	2.02	2.56	7.20	21.90	78.30	92.73	93.25	0.83	2.07	3.34	10.90	33.10	137.45	77.23	76.22
	2.00	0.80	3.99	4.06	5.70	18.50	110.30	68.96	75.53	0.97	1.92	1.65	5.60	17.50	81.43	94.23	99.32	0.74	4.34	4.56	13.00	39.70	110.30	68.96	75.53
	3.00	0.64	5.83	5.39	8.50	25.70	117.80	55.20	51.67	0.84	4.04	3.62	10.30	31.20	115.10	80.55	69.78	0.75	5.24	4.76	10.90	38.04	117.80	55.20	51.67
	4.00	0.88	3.65	3.41	5.10	15.40	137.45	77.23	76.22	0.96	2.02	2.56	7.20	21.90	78.30	92.73	93.25	0.83	5.13	4.65	10.90	33.10	137.45	77.23	76.22
	5.00	0.87	3.75	3.59	4.80	14.60	134.55	74.81	75.08	0.96	2.02	2.57	7.20	21.90	78.00	92.59	93.17	0.83	4.34	4.56	10.80	32.90	134.55	74.81	75.08
	6.00	0.86	3.92	3.92	5.10	15.60	104.55	68.32	73.80	0.96	2.01	1.71	6.00	18.30	80.83	94.51	98.32	0.74	4.33	4.59	13.00	39.60	104.55	68.32	73.80
	7.00	0.78	4.76	4.99	6.60	20.20	139.50	59.74	50.20	0.96	2.12	1.98	6.60	20.00	88.73	90.61	99.37	0.94	5.24	4.76	7.60	33.10	139.50	59.74	50.20
	8.00	0.87	3.79	3.59	4.80	14.60	134.55	74.81	75.08	0.96	2.02	2.57	7.20	21.90	78.00	92.59	93.17	0.83	2.60	2.13	10.80	33.10	134.55	74.81	75.08

Pre-processing; M1 = none, M2 = 1DER, M3 = 2DER, M4 = Log10, M5 = Log10 + SG, M6 = 1DER + SG, M7 = 2DER + SG, M8 = SG.

**Table 5:** PLS-R models developed using spectral data from the benchtop Thermo iS50 and portable Flame-NIR on TQ Analyst.

								Recovery %		
		Preprocessing	R^2^	RMSEC	RMSEP	LOD	LOQ	10	20	30
iS50	Salt	2DER + SG	0.95	2.19	2.39	6.02	18.30	120.55	109.91	72.96
	Starch	None	1.00	0.43	0.43	0.90	2.74	100.00	103.84	101.59
	Sawdust	SG	0.97	1.88	3.00	2.86	8.69	87.27	87.64	89.86
FlameNIR	Salt	None	0.78	4.80	4.71	10.38	31.56	154.80	65.01	70.05
	Starch	None	0.98	1.62	1.92	5.73	17.43	102.67	99.15	92.89
	Sawdust	None	0.95	2.56	2.14	7.41	22.52	181.54	105.92	101.97

**Table 6:** Statistical parameters obtained from PLS-R models developed using The Lab software, from spectral data obtained on the SCiO device.

	Salt			Starch			Sawdust		
Model	R^2^	RMSEC	RMSEP	R^2^	RMSEC	RMSEP	R^2^	RMSEC	RMSEP
Raw	0.793	2.94	2.94	0.971	1.23	1.23	0.887	2.17	2.17
Log	0.798	3.013	3.013	0.967	1.296	1.296	0.886	2.26	2.26
Log SNV	0.881	2.46	2.45	0.966	1.273	1.273	0.881	2.135	2.135
Log SNV 1DER	0.792	3.35	3.35	0.963	1.34	1.34	0.885	2.242	2.241

## Appendix A

**Table A1:** Number of factors determined for each model.

				Thermo iS50		Flame-NIR		SCiO	
Type	ID	Model	Pre-Processing	A	R^2^Xcum	A	R^2^Xcum	A	R^2^Xcum
2-CLASS	M1	PLS-DA	None	4	1.00	5	1.00	1	0.99
	M2	OPLS-DA	None	2 + 2 + 0	1.00	1 + 3 + 0	1.00	1 + 1 + 0	0.91
	M3	PLS-DA	SNV	3	0.99	3	0.83	2	0.95
	M4	OPLS-DA	SNV	2 + 1 + 0	0.99	1 + 2 + 0	0.83	1 + 1 + 0	0.78
	M5	PLS-DA	SNV + 1DER	3	1.00	4	0.91	4	0.81
	M6	OPLS-DA	SNV + 1DER	2 + 1 + 0	1.00	1 + 3 + 0	0.91	2 + 2 + 0	0.80
	M7	PLS-DA	SNV + 1DER + SG	3	0.98	3	0.91	4	0.80
	M8	OPLS-DA	SNV + 1DER + SG	2 + 1 + 0	0.93	1 + 2 + 0	0.91	2 + 2 + 0	1.00
MULTICLASS	M9	PLS-DA	None	2	0.98	4	1.00	2	1.00
	M10	OPLS-DA	None	1 + 1 + 0	0.98	2 + 2 + 0	1.00	1 + 2 + 0	1.00
	M11	PLS-DA	SNV	3	1.00	4	0.91	4	1.00
	M12	OPLS-DA	SNV	1 + 3 + 0	1.00	2 + 2 + 0	0.91	2 + 2 + 0	1.00
	M13	PLS-DA	SNV + 1DER	3	1.00	3	0.99	4	1.00
	M14	OPLS-DA	SNV + 1DER	1 + 2 + 0	1.00	2 + 2 + 0	0.99	2 + 2 + 0	0.81
	M15	PLS-DA	SNV + 1DER + SG	3	0.98	3	0.92	4	0.80
	M16	OPLS-DA	SNV + 1DER + SG	1 + 2 + 0	0.92	2 + 2 + 0	0.95	2 + 2 + 0	0.99

A = Number of PLS factors, R^2^Xcum = Cumulative explained fraction of the variation.
